# Patient Satisfaction with Remote Pre-Anesthesia Assessment Via Telephone

**DOI:** 10.1089/tmr.2024.0067

**Published:** 2025-01-17

**Authors:** Armin Langauer, Gernot Gerger, Sabine Völkl-Kernstock, Maria Kletecka-Pulker, Nikolaus Graf, Aylin Bilir, David M. Baron

**Affiliations:** ^1^ Department of Anaesthesia, Medical University of Vienna, Intensive Care Medicine and Pain Medicine, Division of General Anaesthesia and Intensive Care Medicine, Vienna, Austria.; ^2^ Ludwig Boltzmann Institute for Digital Health and Patient Safety, Ludwig Boltzmann Gesellschaft, Vienna, Austria.

**Keywords:** pre-anaesthesia assessment, patient satisfaction, telemedicine, patient safety

## Abstract

**Background:**

The use of telemedicine can contribute to patient satisfaction, support hygienic concepts by avoiding physical contact when not required, and reduce waiting and travel time. We aimed to evaluate our recently implemented telemedical approach of remote pre-anesthesia assessment.

**Methods:**

We designed a questionnaire to assess patient satisfaction with remote pre-anesthesia assessment procedures, completeness of understanding, and technical feasibility. In total, 250 patients were asked to voluntarily complete the questionnaire after their pre-anesthesia assessment via telephone. Digital anesthesia records were subsequently reviewed for unexpected events and complications to investigate the quality and safety of the approach.

**Results:**

Patients included in our study were 51 years old (median, range 18–85 years), mostly female (58%) and had an American Society of Anesthesiologists (ASA) physical status of 1–3 (22.8%, 56.4%, and 20.8%, respectively). Patient satisfaction was high with ratings of “very good” or “good” in over 90% of all questions related to the pre-anesthesia assessment via telephone. Patient’s evaluation for the use of telemedicine in general also showed a wide acceptance with 84.4% rating the idea as “very good” (55.6%) or “good” (28.8%). Duration of patient-physician interaction positively correlated with age (*p* = 0.005) and ASA status (*p* = 0.003). Upon review of the digital anesthesia records, there were no intraoperative complications or unexpected events related to the remote pre-anesthesia assessment.

**Conclusion:**

Remote pre-anesthesia assessment via telephone is safe, technically feasible, and satisfactorily accepted in selected patients. These results encourage the continuing implementation of telemedical approaches for pre-anesthesia assessment.

## Introduction

Patient satisfaction plays an increasingly important role in the evaluation of medical care.^1–3^ Patients who are satisfied with their medical care are more likely to adhere to recommended therapies and attend their follow-up appointments more regularly. As a consequence, medical procedures can be scheduled more reliably, and patient outcomes can be potentially improved.^2^

In contrast to physiological parameters that can be directly measured, satisfaction is perceived subjectively. As many factors have to be taken into consideration, assessment of satisfaction can be challenging.^1,4^ Surveys are the tool of choice to evaluate patient satisfaction, and several factors have been identified to contribute to reported patient satisfaction.^4^ These include hospital size and cleanliness, staffing (i.e., personnel-to-patient ratio), perceived individualization of care, quality of communication with doctors and nurses, and total waiting time.^3–5^ Recently, the use of telemedicine has been the focus of interest, both regarding its feasibility and its acceptance by patients and medical care providers.^6^

Telemedicine describes, among other applications of the term, the use of various information technology systems to connect patient and provider for consultation visits, assessments, or any other exchange of information needed. Connecting patients and providers via telephone calls, offering online video information, and digital document transmission can reduce waiting times, limit travel to the clinic or office, and help ensure quality of care.^7^ In addition, telemedicine supports hygiene concepts by avoiding unnecessary contacts in common waiting areas, especially with the background of the recent COVID-19 pandemic.

Several studies have already shown the feasibility of telemedicine applications in the setting of pre-anesthesia assessments and the acceptance of this modality by providers and patients.^8–10^ The use of telemedicine tools can positively impact patient satisfaction.^11^ A simple workflow and short waiting and travel time were identified as two important factors contributing to patient satisfaction when using telemedicine applications in a recent survey study.^6^

Therefore, we evaluated the procedure of conducting the pre-anesthesia assessment via telephone call, which was recently adopted at our department. The primary aim of the presented study was to assess patient satisfaction with the procedure. In addition, we evaluated whether or not pre-anesthesia assessment via telephone was associated with any perioperative complications.

## Methods

This study was planned as a prospective, unblinded cohort study using a questionnaire as modality of data collection. A questionnaire (see Supplementary Data S1) was designed to evaluate patient satisfaction with the process of our department’s pre-anesthesia assessment for elective surgical procedures conducted via telephone call. The questionnaire was initially created by the members of the study team affiliated with the Department of Anaesthesia, Intensive Care Medicine and Pain Medicine. The design was based on the procedure of our pre-anesthesia department, the contents of the informed consent sheet and the typical sequence of patient-provider interaction. The drafted questionnaire was then revised, complemented, and validated by psychologists associated with the Ludwig Boltzmann Institute for Digital Health and Patient Safety (Ludwig Boltzmann Gesellschaft, Vienna, Austria).

After obtaining the approval from the local ethics committee (EK number 1688/2018), we enrolled 250 patients who received a pre-anesthesia assessment for elective surgery via telephone call through our pre-anesthesia outpatient clinic. The time span for inclusion was from June 2020 until June 2021. All patients over the age of 18 years, who received a pre-anesthesia assessment for elective surgery over the phone, were eligible for inclusion. Patients who were not able to consent for themselves (e.g., when legal guardianship for adults was in place) and patients who needed a translator to give informed consent were also excluded from this study. We had intended to include an additional group of patients receiving a face-to-face pre-anesthesia evaluation at our outpatient clinic, but ultimately did not because there were relatively few eligible patients during the study period.

All patients enrolled in this study received a pre-anesthesia evaluation according to the clinical standards of our department. Prior to informed consent, individuals had the possibility to obtain information regarding the anesthetic procedures through online videos and educational material. Our department routinely uses the Thieme Compliance Anesthesia Information Sheet and Medical History Questionnaire An 1E (Thieme Media, Stuttgart-Feuerbach, Germany) to obtain complete documentation of the patient’s medical history during our pre-anesthesia evaluation. After the assessment, patients were asked to voluntarily participate in a feedback questionnaire regarding their satisfaction with the services provided, which would take about 10 min to complete. It was explained that the results of the questionnaire would be used for study purposes and quality control.

The questionnaire started with a brief collection of relevant demographic data. In addition to questions regarding satisfaction with our procedures, it contained questions inquiring about the completeness of understanding of the information provided and the technical feasibility of the pre-anesthesia assessment over the telephone. Questions were placed in thematically structured segments of the questionnaire. The questionnaire further evaluated the patient’s perception of being adequately informed about the anesthetic procedure to be performed, and whether the necessary sphere of personal privacy could be preserved during the phone call. Patients were asked to evaluate aspects of the anesthetic assessment on Likert scale ratings from 1 to 5. Possible answers were broken up as follows: 1 for “very good,” 2 for “rather good,” 3 for “moderate,” 4 for “rather bad,” and 5 for “very bad.” Furthermore, the time needed for each individual pre-anesthesia assessment was recorded.

Finally, digital anesthesia records of all patients included in this study were reviewed in the patient data management system to evaluate whether any unexpected complications occurred during the surgical procedure. The sample size was selected based on similarly designed studies found in recent literature.^1,8,9^ After patients were enrolled, the results were anonymized and analyzed statistically. Characteristics of study participants are reported as median, range, and (relative) frequencies. The subjective ratings of the participants are presented as mean values with standard deviations and relative percentages. The interactions between variables were assessed using Pearson’s correlation analysis, one-way analysis of variance, and unpaired *t*-tests, depending on the specific context and requirements of the analysis. Differences were considered statistically significant for an alpha value <0.05. The article was prepared according to STROBE criteria.

## Results

### Characteristics of the study participants

All 250 patients included in this study were eligible for analysis. The summary of patient demographics given in Table 1 shows the distribution of age, gender, American Society of Anesthesiologists (ASA) physical status classification, and the distribution of level of education.

**Table 1.** Characteristics of Study Participants

Variable	Median	Range
Age (years)	51	18–85

Variable	*n*	%
Gender		
Male	105	42.0
Female	145	58.0
Education		
Primary/none	37	14.8
Advanced/vocational	84	33.6
Secondary	64	25.6
Graduate/postgraduate	50	20.0
Not disclosed	15	6.0
ASA classification		
ASA 1	57	22.8
ASA 2	141	56.4
ASA 3	52	20.8

ASA, American Society of Anesthesiologists physical status classification.

### Patient satisfaction

**FIG. 1.**
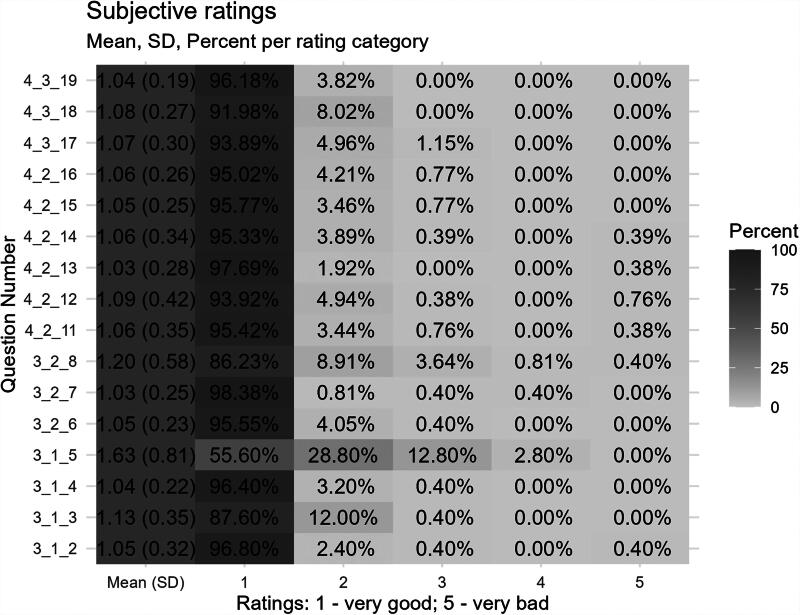
Mean ratings (patient answers) and percent per rating category for every survey question for which ratings apply. For full text questions see Supplementary Data S1.

Patient satisfaction with our procedure was high throughout all survey sections, which focused on satisfaction with patient-provider interaction, completeness of understanding, organization, and technical feasibility (Supplementary Data S1). Figure 1 shows the distribution of ratings from 1 (very good) to 5 (very bad) obtained for each question in the survey.

Ratings of “very good” were reported in over 90% of the majority of questions in the survey (Fig. 1). More mixed results were obtained for the attitude toward the use of telemedicine in general (Question 3_1_5 in Fig. 1) with 84.4% of patients rating the use of telemedicine as either “very good” (55.6%) or “good” (28.8%). In addition, speech intelligibility over the telephone (Question 3_2_8 in Fig. 1) was rated as “very good” by 86.23% and as “good” by 8.91% of participants. Furthermore, patients evaluated the time required to complete the informed consent form (Question 3_1_3 in Fig. 1) with “very good” in 87.6% and “good” in 12.0%.

### Duration of patient-provider interaction

**FIG. 2.**
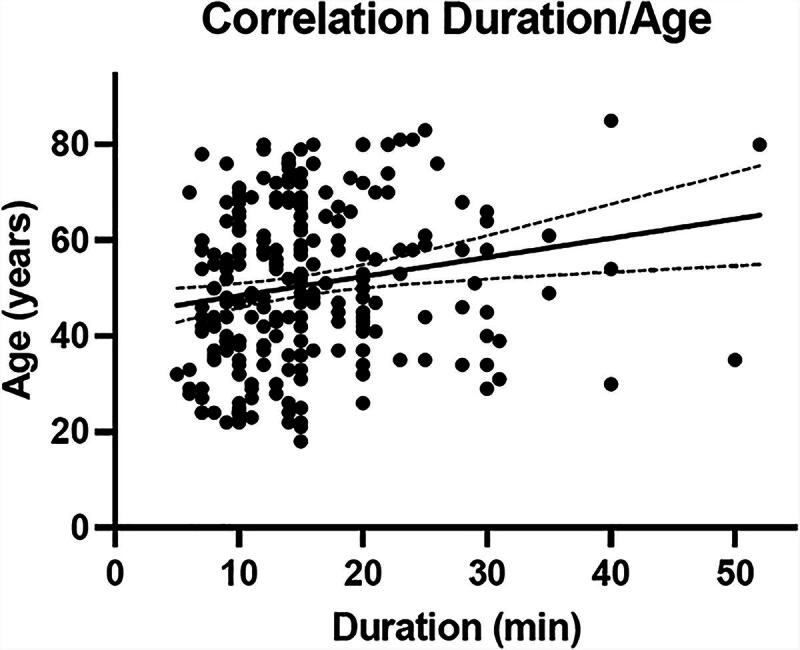
Correlation of patient-provider interaction duration and patient age.

**FIG. 3.**
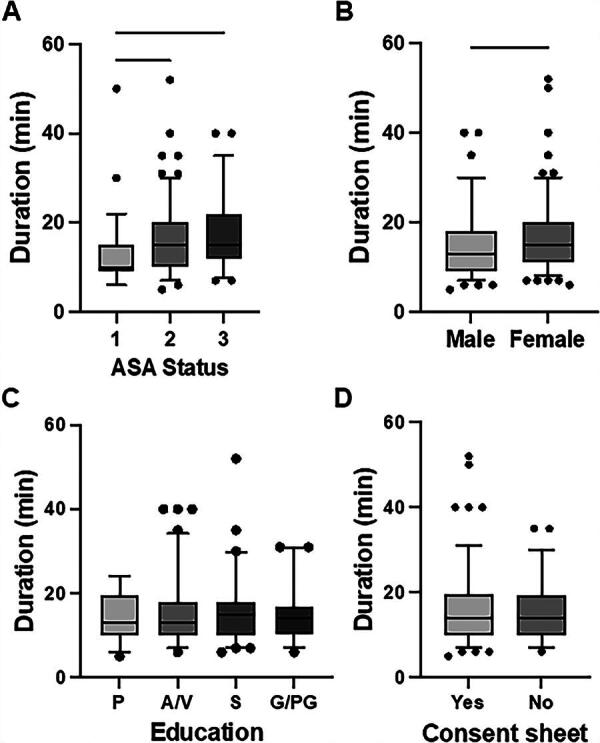
Duration of patient-provider interaction according to **(A)** American Society of Anesthesiologists (ASA) physical status classification classification, **(B)** patient gender, **(C)** patient level of education, and **(D)** receipt of consent sheet prior to pre-anesthesia assessment. P, primary; A/V, advanced/vocational; S, secondary; G/PG, graduate/postgraduate.

The recorded mean duration of patient-physician interaction was 15 min (95% confidence interval 10–18 min). Age positively correlated with time needed to obtain informed consent (*r*^2^ 0.32; *p* = 0.005, Fig. 2). Compared with patients with ASA 1 status (13 [95% confidence interval 11–15] min), longer patient-physician interactions were recorded for patients with ASA 2 (16 [95% confidence interval 14–17] min, *p* = 0.017) and ASA 3 status (18 [confidence interval 15–20] min, *p* = 0.003, Fig. 3A). In addition, patient-physician interaction was longer for female (16 [95% confidence interval 15–17] min) than for male patients (14 [95% confidence interval 13–15] min, *p* = 0.042, Fig. 3B).

Level of education had no influence on the time used for obtaining informed consent in this study (*p* = 0.778, Fig. 3C). Similarly, whether patients had studied the informed consent sheet or additional educational material prior to their interaction with our physicians showed no influence on the duration of patient-physician interaction (*p* = 0.316, Fig. 3D).

### Intraoperative complications

Finally, all digital anesthesia protocols were evaluated for complications and unexpected events. No relevant intraoperative complications (anaesthesiologic and surgical) or unexpected events were identified. Unexpected events included in our patient data management system relate to waiting times on the part of the surgical team, failure to establish peripheral venous access on the ward, unavailability of IT services due to maintenance, and procedural difficulties.

## Discussion

In this study, we evaluated patient satisfaction after pre-anesthesia assessment via telephone. Throughout all categories, satisfaction was high, with 84.4% of all participants evaluating the use of telemedicine as “very good” or “good.” These results suggest a wide acceptance of the method. In addition, we did not identify any intraoperative complications or unexpected events upon review of the digital patient files.

The data obtained in this study are consistent with a recent evaluation of telemedicine during the COVID-19 pandemic in Austria. Around one third of those who had contacted a doctor during that period did so through telemedical methods. Satisfaction with telehealth services was generally high, especially among those with prior experience, with a 90% satisfaction rate reported. The most common method of communication was telephone. Overall, the majority (53%) of respondents perceived significant advantages to telemedicine.^6^

In the current study, we further demonstrated the feasibility of pre-anesthesia assessment via telephone. Almost all patients (97%) stated they were able to use the telephone without assistance, and 95% reported that they understood the physician either “very well” or “well.” Previous studies have reported the feasibility, safety, and high levels of patient satisfaction associated with telephone pre-anesthesia assessment. These findings were documented both prior to the COVID-19 pandemic and after its onset.^8,12–15^

Subgroup analyses revealed that the duration of patient-physician interaction varied, with older patients requiring more time for informed consent compared with younger patients, females needing more time than males, and patients with relevant systemic diseases, as indicated by their ASA physical status classification, requiring extended interaction periods. Several factors might contribute to this finding. Older patients often have multiple comorbidities, which can complicate treatment decisions. Physicians need to spend more time discussing these conditions and their potential impact on treatment options. Furthermore, older patients may have decreased cognitive function or other factors that affect their ability to understand medical information. Finally, these patients may have unique concerns and preferences related to their age and health status. Physicians thus need to address these concerns and accommodate preferences during the informed consent process, which can require additional time. Despite the increased time required for patients with higher comorbidity, a recent study showed that the use of telemedical pre-anesthesia assessment is feasible and safe for ASA 3 and ASA 4 patients, which is consistent with our results.^16^

The level of education completed by the patient (primary, secondary, vocational, higher, or none) was not significantly associated with the duration of patient-physician interaction. Handing out information sheets about anesthetic procedures by the referring surgical department was done inconsistently. However, receiving an information sheet showed no significant impact on the duration of patient-physician interaction and one third of patients who received an information sheet and link to information videos reported that they were either not interested or did not find the time to make use of the opportunity.

No unexpected events or complications in relation to the pre-anesthesia assessment were recorded during the following surgery. Recorded events included organizational delays on the side of the surgical departments, technical difficulties, and procedure-related events. These data indicate that pre-anesthesia assessment via telephone does not reduce patient safety when compared with in-person assessment. These results are in line with comparable recent studies on the use of telemedical pre-anesthesia assessment.^12,16,17^ A report on pre-operative anxiety compared patient-reported anxiety levels after face-to-face pre-anesthesia assessment and assessment via telephone call.^18^ No difference in pre-operative anxiety was found, which further supports the previously reported high levels of patient safety and satisfaction with this method.

Our evaluation was limited due to a missing control group. In the original study design, an equally sized control group consisting of patients who completed their pre-anesthesia assessment in physical attendance was planned. Due to a relatively small sample size of these patients during the inclusion period, this cohort was not included in our analysis. The ongoing COVID-19 pandemic and associated strict hygiene regulations demanded avoidance of physical contact wherever reasonably possible. Consequentially, only patients who had to be present in-house due to ongoing illness or those regarded as complicated cases were seen personally in our pre-anesthesia department. This relatively small patient group showed significant differences in ASA physical classification status and time needed for the assessment due to their non-trivial clinical situation and was therefore not analyzed to avoid further bias. Another limitation in this study arises from excluding patients who needed a translator for their pre-anesthesia assessment and therefore no statement can be made about patient satisfaction in this subgroup. It was chosen to exclude these patients to preserve comparability of the analyzed group since the inclusion of a translator is expected to significantly impact the time needed for the assessment, which is all the more important since waiting time has been shown to be a contributor to overall satisfaction.^4,5^ This group, however, was analyzed in a separate study.^19^

## Conclusion

We have conducted an in-depth analysis of patient satisfaction regarding our recently introduced pre-anesthesia assessment via telephone, and have observed marked patient satisfaction with this method. Our investigation further supports the technical feasibility and safety of pre-anesthesia assessment via telephone. Moreover, the emphasis on hygiene, particularly in light of recent global health concerns, emerges as a significant benefit of telemedical approaches to pre-anesthesia assessments.

## Abbreviations Used

ASA: American Society of Anesthesiologists
COVID-19: Corona Virus Disease-19
