# Supplementary material for: Patient Satisfaction with Remote Pre-Anesthesia Assessment Via Telephone
Source: Telemed Rep. 2025 Jan 17;6(1):27–33. doi: 10.1089/tmr.2024.0067 (PMC11839509; doi:10.1089/tmr.2024.0067)
Supplement: Supplementary Data S1 [file tmr.2024.0067_supp_datas1.pdf]

Questionnaire patient satisfaction pre-anaesthesia assessment

1. Demographic data

Age	_____ years	
Gender	male ◯	female ◯
Have you ever had surgery?	Yes ◯	No ◯
Education (highest completed level)	No graduation ◯ Compulsory school ◯<br>Vocational school ◯ Grammar school ◯<br>University degree ◯	
ASA-classification	1 ◯ 2 ◯ 3 ◯ 4 ◯	

2. Face-to-face assessment

Duration of assessment: **Start** **End**

2.1. Reception at front desk

How easy was it to find the front desk and waiting area?

1 ◯ very good	2 ◯ rather good	3 ◯ moderate	4 ◯ rather bad	5 ◯ very bad

Was all information provided at the front desk clear and comprehensible?

1 ◯ very good	2 ◯ rather good	3 ◯ moderate	4 ◯ rather bad	5 ◯ very bad

How did you perceive the waiting time at the front desk?

1 ◯ very good	2 ◯ rather good	3 ◯ moderate	4 ◯ rather bad	5 ◯ very bad

How satisfied are you with the procedure at the front desk?

1 ◯ very good	2 ◯ rather good	3 ◯ moderate	4 ◯ rather bad	5 ◯ very bad

2.2. Consent Form

How would you rate the comprehensibility of the <u>information</u> on the informed consent form?

1 ◯ very good	2 ◯ rather good	3 ◯ moderate	4 ◯ rather bad	5 ◯ very bad

How would you rate the comprehensibility of the <u>questions</u> on the informed consent form?

1 ◯ very good	2 ◯ rather good	3 ◯ moderate	4 ◯ rather bad	5 ◯ very bad

How would you rate the time required for filling in the informed consent form?

1 ◯ very good	2 ◯ rather good	3 ◯ moderate	4 ◯ rather bad	5 ◯ very bad

Was it generally easy to fill out the informed consent form?

1 ◯ very good	2 ◯ rather good	3 ◯ moderate	4 ◯ rather bad	5 ◯ very bad

3. Remote assessment

3.1. Procedure of the assessment via telephone/video

Were you able to operate the communication device without assistance?

yes ◯	no ◯

Were you sufficiently informed about the purpose and process of the call up front?

1 ◯ very good	2 ◯ rather good	3 ◯ moderate	4 ◯ rather bad	5 ◯ very bad

How would you rate the time required to complete the informed consent form?

1 ◯ very good	2 ◯ rather good	3 ◯ moderate	4 ◯ rather bad	5 ◯ very bad

Overall, are you satisfied with the process of your telephone call?

1 ◯ very good	2 ◯ rather good	3 ◯ moderate	4 ◯ rather bad	5 ◯ very bad

How do you generally evaluate the use of telemedical applications (such as telephone, computer, tablet) when communicating with a physician?

1 ◯ very good	2 ◯ rather good	3 ◯ moderate	4 ◯ rather bad	5 ◯ very bad

3.2. Consent form

Was the information you received phrased comprehensibly?

1 ◯ very good	2 ◯ rather good	3 ◯ moderate	4 ◯ rather bad	5 ◯ very bad

Were questions phrased comprehensibly (e.g. regarding your medical history)?

1 ◯ very good	2 ◯ rather good	3 ◯ moderate	4 ◯ rather bad	5 ◯ very bad

Were you able to understand the physician clearly over the phone?

1 ◯ very good	2 ◯ rather good	3 ◯ moderate	4 ◯ rather bad	5 ◯ very bad

4. General

4.1. Information previous to the assessment

Did you receive the informed consent sheet prior to your anaesthesia assessment?

Did you watch the video information regarding the anaesthesia?

If "no", why not?

4.2. Interaction with the physician

How did you perceive the conversation with your anaesthesiologist?

1 ◯ very good	2 ◯ rather good	3 ◯ moderate	4 ◯ rather bad	5 ◯ very bad

Do you feel sufficiently informed about the anaesthetic procedure and possible risks?

1 ◯ very good	2 ◯ rather good	3 ◯ moderate	4 ◯ rather bad	5 ◯ very bad

Were your questions answered satisfactorily?

1 ◯ very good	2 ◯ rather good	3 ◯ moderate	4 ◯ rather bad	5 ◯ very bad

If "3-5", what questions remained unsolved?

____

Were your concerns regarding your pending anaesthetic procedure addressed sufficiently?

1 ◯ very good	2 ◯ rather good	3 ◯ moderate	4 ◯ rather bad	5 ◯ very bad

Did you have enough time to discuss your medical history during this call?

1 ◯ very good	2 ◯ rather good	3 ◯ moderate	4 ◯ rather bad	5 ◯ very bad

Do you feel adequately informed about the schedule leading up to surgery and how to prepare for your upcoming anaesthetic procedure (e.g. regarding pre-op fasting, obtaining medical reports)?

1 ◯ very good	2 ◯ rather good	3 ◯ moderate	4 ◯ rather bad	5 ◯ very bad

4.3. General impression

How satisfied are you with the overall process of the pre-anaesthesia assessment?

1 ◯ very good	2 ◯ rather good	3 ◯ moderate	4 ◯ rather bad	5 ◯ very bad

How do you evaluate the amount of time you invested in the pre-anaesthesia assessment?

1 ◯ very good	2 ◯ rather good	3 ◯ moderate	4 ◯ rather bad	5 ◯ very bad

Where your expectations regarding privacy met?

1 ◯ very good	2 ◯ rather good	3 ◯ moderate	4 ◯ rather bad	5 ◯ very bad
